# IL-10 Inhibits the NF-κB and ERK/MAPK-Mediated Production of Pro-Inflammatory Mediators by Up-Regulation of SOCS-3 in *Trypanosoma cruzi*-Infected Cardiomyocytes

**DOI:** 10.1371/journal.pone.0079445

**Published:** 2013-11-18

**Authors:** Eugenia Hovsepian, Federico Penas, Sofía Siffo, Gerardo A. Mirkin, Nora B. Goren

**Affiliations:** Instituto de Microbiología y Parasitología Médica (IMPaM, UBA-CONICET), Facultad de Medicina, Universidad de Buenos Aires, Buenos Aires, Argentina; Karolinska Institutet, Sweden

## Abstract

*Trypanosoma cruzi* (*T. cruzi*) infection produces an intense inflammatory response which is critical for the control of the evolution of Chagas’ disease. Interleukin (IL)-10 is one of the most important anti-inflammatory cytokines identified as modulator of the inflammatory reaction. This work shows that exogenous addition of IL-10 inhibited ERK1/2 and NF-κB activation and reduced inducible nitric oxide synthase (NOS2), metalloprotease (MMP) -9 and MMP-2 expression and activities, as well as tumour necrosis factor (TNF)-α and interleukin (IL)-6 expression, in *T. cruzi*-infected cardiomyocytes. We found that *T*. *cruzi* and IL-10 promote STAT3 phosphorylation and up-regulate the expression of suppressor of cytokine signalling (SOCS)-3 thereby preventing NF-κB nuclear translocation and ERK1/2 phosphorylation. Specific knockdown of SOCS-3 by small interfering RNA (siRNA) impeded the IL-10-mediated inhibition of NF-κB and ERK1/2 activation. As a result, the levels of studied pro-inflammatory mediators were restored in infected cardiomyocytes. Our study reports the first evidence that *T. cruzi* up- regulates SOCS-3 expression and highlights the relevance of IL-10 in the modulation of pro-inflammatory response of cardiomyocytes in Chagas’ disease.

## Introduction

Chagas’ disease is a chronic and systemic infection caused by the protozoan parasite *Trypanosoma cruzi*. The acute phase of infection is characterized by the presence of parasites in the host bloodstream and diverse tissues. However, the heart is one of the principal targets of this disease, causing serious cardiac alterations in the acute and chronic phases. Our group and others have previously described that during the acute phase of the disease, there is induction and high-level expression of pro-inflammatory cytokines as well as of inducible nitric oxide synthase (NOS2) [1]–[4], prostaglandins [5], [6] and chemokines [7]. A number of cardiac pathophysiological conditions, including myocardial infarction and ischemia-reperfusion injury leading to heart failure, are associated with activation of inflammatory mediators in the heart [3], [4]. Expression of pro-inflammatory cytokines, such as tumor necrosis factor-α (TNF-α), interleukin-1 (IL-1) and interleukin-6 (IL-6) and anti-inflammatory cytokine IL-10, mediate homeostasis within the heart in response to injury. However, a sustained expression of pro-inflammatory mediators at sufficiently high concentrations could lead to an adverse outcome, including heart failure [8]–[10]. The inflammatory response is a key component of host defense, but excessive inflammation such as that occurring in several inflammatory diseases can be deleterious or even fatal. Therefore, inflammation must be tightly regulated. Interleukin-10 (IL-10) is a key inhibitor of many aspects of the inflammatory response. This cytokine is a potent anti-inflammatory cytokine, a strong deactivator of monocytes and suppressor of various pro-inflammatory mediators [11], [12]. However, the therapeutic effects of systemic IL-10 on inflammation-mediated cardiac dysfunction and remodeling, as well as the molecular signaling pathways that explain these effects, remain to be elucidated. Engagement of the IL-10 receptor has been shown to involve the JAK-STAT signaling pathway inducing the activation of STAT1, STAT3, and, in some cells, STAT5 [13]. STAT3 can be activated not only by IL-10 signaling, but also by many other cytokines like IL-6, transcription factors and oncogenic proteins [14]–[16]. In response to cytokine signaling, STAT3 activates certain genes whose products were identified in the mid-1990s as suppressors of cytokine signaling (SOCS) [17]. Some authors suggest that, among them, SOCS-1 and SOCS-3 are induced only by IL-10-mediated signaling [18], [19]. It has been described that SOCS-3 exerts its anti-inflammatory effects by inhibition of the activation of the NF-κB pathway. In fact, Nair et al. [20] demonstrated that the PPE18 protein of *Mycobacterium tuberculosis* inhibits LPS-induced IL-12 and TNF-α production by blocking nuclear translocation of p50, p65 NF-κB, and c-rel transcription factors in macrophages. These authors found that PPE18 up-regulates the expression as well as tyrosine phosphorylation of SOCS-3, and that phosphorylated SOCS-3 physically interacts with the IκBα–NFκB/rel complex, inhibiting phosphorylation of IκBα. It has been described that SOCS-2 is expressed during *T. cruzi* infection and it is postulated that it may therefore participate in the pathogenesis of Chagas’ disease by modulating heart damage [21]. However, the significance of SOCS-3 in the modulation of inflammatory response in *T. cruzi* infection has not been studied.

The aim of this work was to analyse whether the addition of exogenous IL-10 modulates inflammatory mediators produced by *T. cruzi-*infected cardiomyocytes and the molecular events leading to such regulation. Our results show that IL-10 leads to STAT3 activation and up-regulation of SOCS-3. Moreover, specific SOCS-3 knock-down by SOCS-3-siRNA revealed that IL-10 anti-inflammatory effect involves inhibition of NF-κB and ERK signaling with the consequent downregulation of cytokines, NOS2 and matrix metalloproteinases (MMPs) by means of STAT3/SOCS-3 activation pathway.

## Materials and Methods

### Mice and Infection

Twenty one days-old male CF1 mice used in this study were infected by intraperitoneal route with 1×10^5^ bloodstream trypomastigotes of the lethal pantropic/reticulotropic RA strain of *Trypanosoma cruzi*, as previously described [22]. All animal experiments were approved by the Institutional Committee for the Care and Use of Laboratory Animals (SICUAL, University of Buenos Aires School of Medicine). The protocols are in accordance with guidelines of the Argentinean National Administration of Medicines, Food and Medical Technology (ANMAT), Argentinean National Service of Sanity and Agrifoods Quality (SENASA) and also based on the US NIH Guide for the Care and Use of Laboratory Animals.

### Neonatal Mouse Primary Cardiomyocytes Culture

One- to three-day old neonatal CF-1 mice were euthanized by cervical dislocation. The hearts were aseptically removed and kept in Hanks’ balanced salt solution without Ca^2+^ and Mg^2+^ (HBSS 1) (in g/1∶0.4 KCl, 0.06 KH_2_PO_4_, 8.0 NaCl, and 0.05 Na_2_HPO_4_, pH 7.4) on ice. The tissues were washed three times and minced into small fragments. The cells were dissociated with trypsin (Sigma-Aldrich Co., St. Louis, USA) (0.25% wt/vol in HBSS 1) at 37°C. The released cells obtained after the first digestion were discarded, whereas the cells from subsequent digestions were added to an equal volume of cold HBSS 1 with Ca^2+^ and Mg^2+^: HBSS 2 (in g/l: HBSS 1 plus 0.14 CaCl_2_, 0.047 MgCl_2_, 0.049 MgSO_4_, 0.35 NaHCO_3_, and 1.0 D-glucose, pH 7.4) until all cardiac cells were isolated [23]. The resulting cell suspension was centrifuged at 200 g for 8 min, and the cells were resuspended in DMEM: M-199 medium (Gibco™, Grand Island, N.Y., USA) (4∶1, vo/vol) supplemented with 10% Foetal Calf Serum (FCS) (PAA Laboratories GmbH, Haidmannweg, Austria) and antibiotics (50 µg/ml of penicillin, streptomycin, and gentamicin). To exclude non-muscle cells, the isolated cells were first plated in tissue culture dishes at 37°C for 2 h under a water-saturated atmosphere of 5% CO_2_. The non-adherent cells were then collected and plated at a density of 1.0×10^5^ cells cm^2^ in 10% FCS-DMEM: M-199 and antibiotics. More than 90% of cells were cardiomyocytes as detected by immunostaining with antibody to α-actinin (Sigma-Aldrich Co., St. Louis, USA). After 48 h, the culture medium was changed to 1% FCS-DMEM: M-199 4:1 (vol/vol) with or without antibiotics. The cells were infected for the indicated periods of time with *Trypanosoma cruzi* RA strain, treated with IL-10 (BD Pharmigen™ CA, USA), STAT3-specific Inhibitor V stattic [24] (Santa Cruz Biotechnology Inc., CA, USA) or transfected with SOCS-3-siRNA (Santa Cruz Biotechnology Inc., CA, USA). The solvent for IL-10 was a sterile aqueous solution containing 2.5 mg/ml BSA, and the solvent for stattic was DMSO. SOCS-3 siRNA was resuspended in RNAse-free water. After different treatments cell viability was examined by Trypan blue dye exclusion test.

### Infection of Myocardial Cells by *Trypanosoma cruzi*

Bloodstream trypomastigotes used to infect culture cardiomyocytes were obtained by cardiac puncture, from euthanised 21 days-old male CF-1 mice infected 7 days before with trypomastigotes of the RA strain of *T. cruzi*. Briefly, blood diluted 1∶2 in DMEM was centrifuged at 400 g at room temperature and then left at 37°C for 1 h in a water bath. Cardiomyocytes were infected at a 5∶1 parasite:cell ratio in six- (2×10^6^ cell/well) or 24- (5×10^5^ cell/well) well polystyrene plates (containing round glass coverslips). After 3 h, the infected cultures were washed with 1% FCS-DMEM:M-199 fresh medium five times to remove free parasites and then the cells were cultured for different periods (24, 48 or 72 h) under the same conditions.

### NO Measurement

To determine the amount of NO released into the medium, nitrate was reduced to nitrite, and this was measured spectrophotometrically by the Griess reaction, as previously described [25]. The absorbance at 540 nm was compared with a standard curve of NaNO_2_. *In situ* NO synthesis was performed in cardiomyocytes loaded with 4-Amino-5-Methylamino-2′,7′-Difluorofluorescein Diacetate (DAF-FM), following the controls and recommendations of the supplier (Molecular Probes,Eugene, OR, USA). DAF-DM is a non-fluorescent compound that reacts with NO to form fluorescent benzotrizole with excitation/emission at 495/515 nm.

### Quantitative Real-time RT-PCR (Q-RT-PCR)

Total RNA was extracted from frozen cells using Trizol reagent (Life Technologies, Inc., CA, USA). Total RNA was reverse transcribed using Expand Reverse Transcriptase (Promega Corporation, Wisconsin, USA). Q-RT-PCR was performed using SyBr Green PCR kit (PE Applied Biosystems Inc., CA, USA) in an Applied Biosystems 7500 sequence detector. Primer sequences were: MMP-2 forward: 5′-CGGAGATCTGCAAACAGGACA-3′, reverse: 5′-CGCCAAATAAACCGGTCCTT-3; MMP-9 forward: 5′-CAGACCAAGGGTACAGCCTGTT-3′, reverse: 5′-AGTGCATGGCCGAACTC-3′; TNF-α forward: 5′-ATGAGCACAGAAAGCATGATC-3′, reverse: 5′-TACAGGCTTGTCACTCGAATT-3′; IL-6 forward: 5′-TGATGCACTTGCAGAAAACAA-3′, reverse: 5′-GGTCTTGGTCCTTAGCCACTC-3′. SOCS-3 forward: 5′-CCTTTGACAAGCGGACTCTC-3′, reverse: 5′-GCCAGCATAAAAACCCTTCA-3′. All samples were analysed in the same run with 18S rRNA amplification for normalization: forward: 5′-AACACGGGAAACCTCACCC-3′, and reverse: 5′-CCACCAACTAAGAACGGCCA-3′. PCR parameters were 50°C for 2 min, 94°C for 2 min, and 40 cycles of 94°C for 30 s, 60°C (for MMP-9, MMP-2, TNF-α, SOCS-3 and 18S) or 54°C (for IL-6) for 1 min. Quantification was calculated using the comparative threshold cycle (Ct) method and efficiency of the RT reaction (relative quantity, 2^−ΔΔCt^). The replicates were then averaged and fold induction was determined, considering the value at zero time as 1 [23].

### Small Interfering RNA (siRNA)

Cardiomyocytes were cultured up to 30–50% of confluence in DMEM: M-199 medium containing 5% FCS without antibiotics for 24 h. After that, cells were transfected with SOCS-3 siRNA that targets SOCS-3 mRNA, following the manufacturer’s instructions (Santa Cruz Biotechnology Inc., CA, USA). Transfections were performed with Oligofectamine (Life Technologies, Inc., CA, USA) as specified by the manufacturer. Assays for gene activity were performed at 24 and 72 h post transfection. The impact of SOCS-3-siRNA interference on SOCS-3 mRNA was evaluated by Q-RT-PCR.

### Measurement of IL-10 Production

IL-10 concentrations were measured in cell culture supernatants by ELISA. The procedures for ELISA kits (BD Biosciences OptEIA) were performed according to the manufacturer’s instructions. The reaction was detected by peroxidase-conjugated Streptavidin followed by a substrate mixture that contained hydrogen peroxide as a substrate and ABTS (Sigma Aldrich Co., St. Louis, USA) as a chromogen. The absorbance measured at 405 nm was compared with IL-10 standard curve.

### Preparation of Cytosolic, Nuclear and Total Protein Extracts and Western Blot

Cultured cells were washed with PBS and scraped off the dishes with 100 µl of buffer A (10 mmol/l HEPES; pH 7.9, 1 mmol/l EDTA, 1 mmol/l EGTA, 10 mmol/l KCl, 1 mmol/l DTT, 0.5 mmol/l phenylmethyl sulfonyl fluoride, 40 µg/ml leupeptin, 2 µg/ml tosyl-lysyl-chloromethane, 5 mmol/l NaF, 1 mmol/l NaVO_4_, 10 mmol/l Na_2_MoO_4_), and NP-40 was added to reach 0.5% (vol/vol). After 15 min at 4°C, the tubes were gently vortexed for 10 s, and cytosolic extracts were collected by centrifugation at 13,000 g for 30 s. The supernatants were stored at −20°C (cytosolic extracts). Nuclear proteins were obtained by centrifugation at 13,000 g for 5 min, and a liquots of the supernatant (nuclear extracts) were stored at −80°C.

Total protein extracts were obtained after washing cells with PBS and adding OGP (Sigma-Aldrich Co., St. Louis, USA).) lysis buffer (90 µl/dish). Then, the dishes were kept on ice for 30 min with swirling and the scrapped cells were centrifuged at 7,000 g at 4°C for 10 min. The supernatant was transferred to a clean tube and stored at −20°C. Protein concentration was determined by Bradford method using Bio-Rad Protein Assay (BIO-RAD CA., USA) reagent and bovine serum albumin (BSA) (Sigma-Aldrich Co., St. Louis, USA) as pattern protein [25].

For Western blot analysis, cytosolic or total proteins were boiled in Laemmli sample buffer, and equal amounts of protein (40–50 µg) were separated by 10–12% SDS-PAGE. The gels were blotted onto a Hybond-P membrane (GE Health-care, Madrid, Spain) and incubated with the following antibodies (Abs): anti-NOS2, anti- IκB-α, anti-p65, anti-Sp1 (Santa Cruz Biotechnology, CA, USA), anti-P-ERK1/2, anti-P-Tyr705-STAT3 (Cell Signalling Technology Inc., MA, USA) and anti-α-actin (Sigma-Aldrich Co., St. Louis, USA). The blots were revealed by enhanced chemoluminiscence (ECL) in an Image Quant 300 cabinet (GE Healthcare Biosciences, PA, USA) following the manufacturer instructions. Band intensity was analysed using NIH-image J program [23].

### Peritoneal Macrophages Isolation and Activation

Macrophages were obtained by washing the peritoneal cavity of BALB/c mice with 5 ml of Dulbecco’s Modified Eagle Medium (D-MEM; Invitrogen Life Technologies, Grand Island, NY) supplemented with 10% of heat-inactivated FCS. Cells were left to adhere to plastic for 2 h at 37°C. After washing twice with phosphate buffer saline (PBS), adherent cells were scraped and suspended in culture medium. Cell viability (>95%) was assessed by Trypan blue dye exclusion test. For activation macrophages were treated with 1 µg/ml of LPS from *Eschericchia coli* (Sigma–Aldrich, St. Louis, MO) for 24 h.

### Zymography

MMP activity in cell culture supernatants was measured using gelatin in-gel zymography. Culture media were subjected to a 7.5% SDS-PAGE, in which 1 mg/ml gelatin (type A from porcine skin) had been incorporated. Following electrophoresis, gels were washed in 30% Triton X-100 for 60 min to remove SDS. Then, the gels were incubated in 50 mM Tris buffer pH 7.4, containing 0.15 mM NaCl and 30 mM CaCl_2_, for 36 h at 37°C. Gels were stained with Coomassie blue and then destained with 10% acetic acid and 30% methanol in water. The areas of proteolytic activity appeared as negative-stained bands in the dark background. The identities of MMPs were based on their molecular weights and a positive internal control (activated peritoneal macrophages) was run in each gel to allow the standardization of the results obtained in the different zymograms. Fifty µg total supernatant protein (Bradford) was loaded onto each lane. A pre-stained molecular weight marker (Bio-Rad, USA) was used to assess the MW of the bands. After staining with Coomassie blue and destaining, clear and digested regions representing MMPs activity were quantified by densitometry using NIH-image J program.

### Immunofluorescence and Digital Image Analysis

Parasite staining and digital imaging were performed as previously described by Hovsepian et al., with minor modifications [3]. Briefly, myocardial cells grown on round glass coverslips were blocked with 3% normal goat serum in PBS. The percentage of infected cells and the number of amastigotes *per* cell were determined by analyzing the presence of intracellular amastigotes by immunofluorescence. For this purpose, a rabbit polyclonal IgG directed to *T. cruzi* and a FITC-labelled goat anti-rabbit IgG (Sigma–Aldrich) were used at 1∶200 dilutions (determined by titration). Myocardial cells nuclei were stained with DAPI (300 nM in PBS). At least 30 random microscopic fields (400X) and 1000 cells *per* culture were acquired using a Spot RT digital camera attached to an Eclipse 600 fluorescence microscope (Nikon Inc., USA). Cell quantification was performed with the ImageJ open source software developed at the NIH, USA.

### Statistical Analysis

Results are the average±SD of at least three separate experiments. *P* values were determined using Student’s *t*-test or Tukey test for nonparametric values. *P*<0.05 was considered to be statistically significant. For percentages of infected cells and number of amastigotes per cell, the differences were established for a significance level of α = 0.05 by one-way ANOVA and the Tukey post-test. NO levels were quantified by integrating the area of fluorescence, under the assumption that pixel density is directly proportional to NO production. The differences were established for a significance level of α = 0.05 by one-way ANOVA and Bonferroni test for multiple comparison. Statistical analyses were performed using GraphPad Prism 4.0 program.

## Results

### Exogenous IL-10 Attenuates NF-κB and ERK Activity in *T. cruzi*-infected Cardiomyocytes

**Figure 1:**
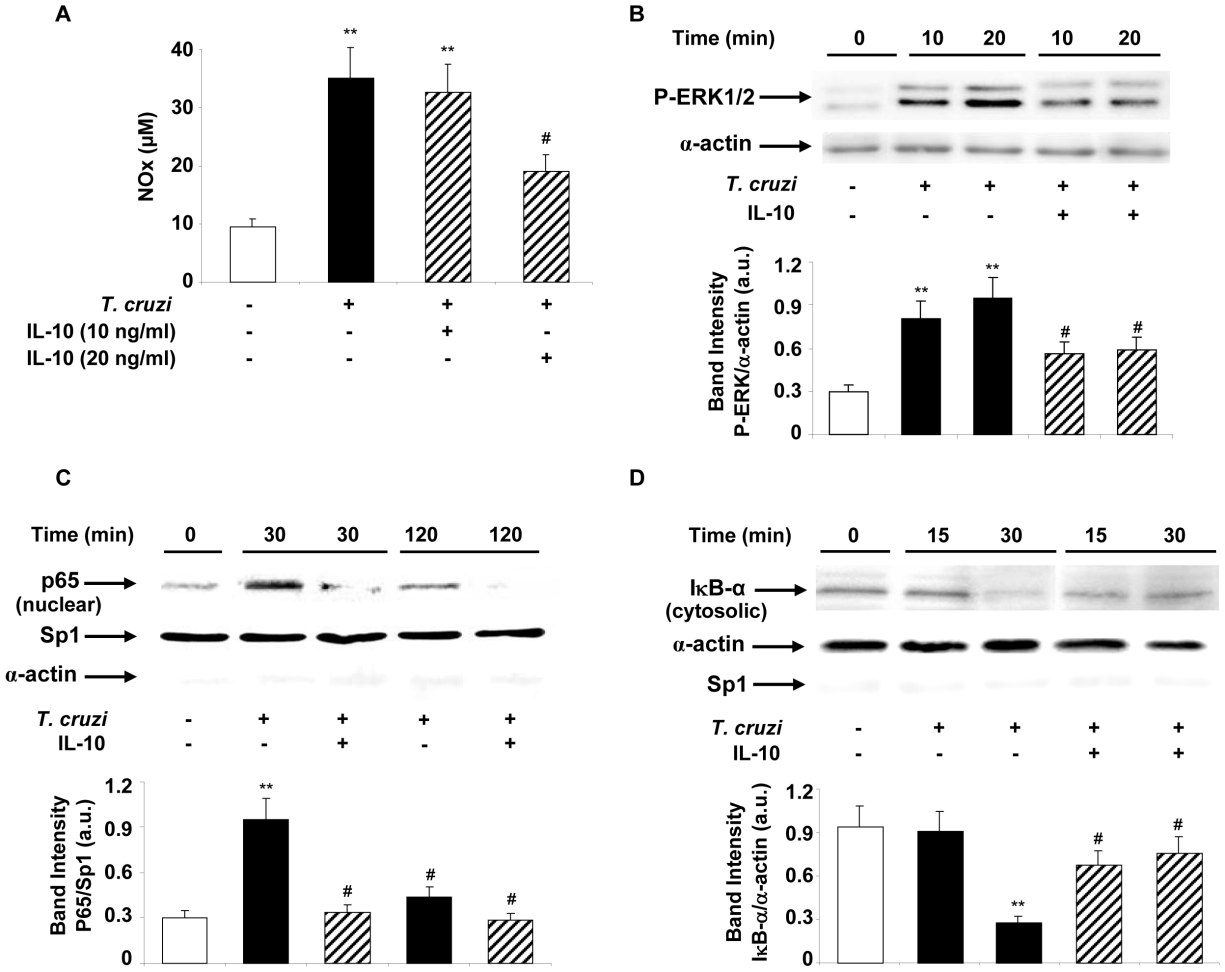
IL-10 inhibits cardiomyocytes inflammatory response induced by *T. cruzi* infection. (A) Cardiomyocytes were pre-treated with IL-10 (10 ng/ml or 20 ng/ml) for 15 min before *T. cruzi* infection (with parasite:cell ratio 5∶1) and NO levels were quantified by Griess reaction in the culture media after 48 h (n = 3). (B) Neonatal cardiac cells were infected for 10 and 20 min or pre-treated for 15 min with 20 ng/ml of IL-10 and cytosolic extracts were prepared. The levels of P-ERK1/2-MAPK were determined by Wb with specific antibodies. (C) Cells were pre-treated with IL-10 in the same conditions as in B and infected with *T. cruzi* during 30 or 120 minutes for p65 nuclear detection or (D) pre-treated with IL-10 and infected for 15 or 30 min for cytosolic detection of IκB-α. For NO measurement, results are expressed as mean±SD (n = 4). For B, C and D results show a representative experiment of three performed and protein levels were normalized against α-actin (B and D) and Sp1 (C). Mean±SD (n = 3). **p<0.01 vs. control cells; ^#^p<0.05 vs. *T. cruzi* infected cells.

Recombinant mouse IL-10 cytokine inhibited *T. cruzi*-induced pro-inflammatory mediators like NO in a concentration-dependent manner in infected cardiomyocytes (Figure 1A). IL-10 at a concentration of 20 ng/ml significantly inhibited NO production. Therefore, 20 ng/ml was used for all our subsequent experiments.

The transcription factor NF-κB and the ERK/MAPK signaling pathways are known to participate in the induction of proinflammatory genes. For that reason, we evaluated if their activities were modified upon IL-10 addition to infected cardiomyocytes. Since IL-10 was found to inhibit NO in *T. cruzi*-infected cardiomyocytes (Figure 1A), we next examined ERK/MAPK activation and the nuclear levels of p65-NF-κB protein in these cells by Western blotting, using specific antibodies against these transcription factors. Cultured cardiomyocytes were incubated with IL-10 (20 ng/ml) and, after *T. cruzi* infection, ERK1/2 phosphorylation was assessed. IL-10 significantly inhibited ERK1/2 phosphorylation (Figure 1B). Nuclear levels of p65 were also decreased in infected and IL-10-treated cardiomyocytes as compared with the untreated infected cells (Figure 1C). It is known that the NF-κB inhibitor IκBα interacts and sequesters p65 among other proteins of the NF-κB complex in the cytoplasm, preventing its nuclear translocation and the transcription of the target genes. Therefore, we next determined whether IL-10 modifies IκBα cytoplasmic levels in infected cardiomyocytes. Figure 1D shows that IκBα remained in the cytoplasm of IL-10-treated infected cardiomyocytes similarly to the control cells, indicating inhibition of NF-κB activity.

### *T. cruzi* and Exogenous IL-10 Promote STAT3 Activation and SOCS-3 Expression

**Figure 2:**
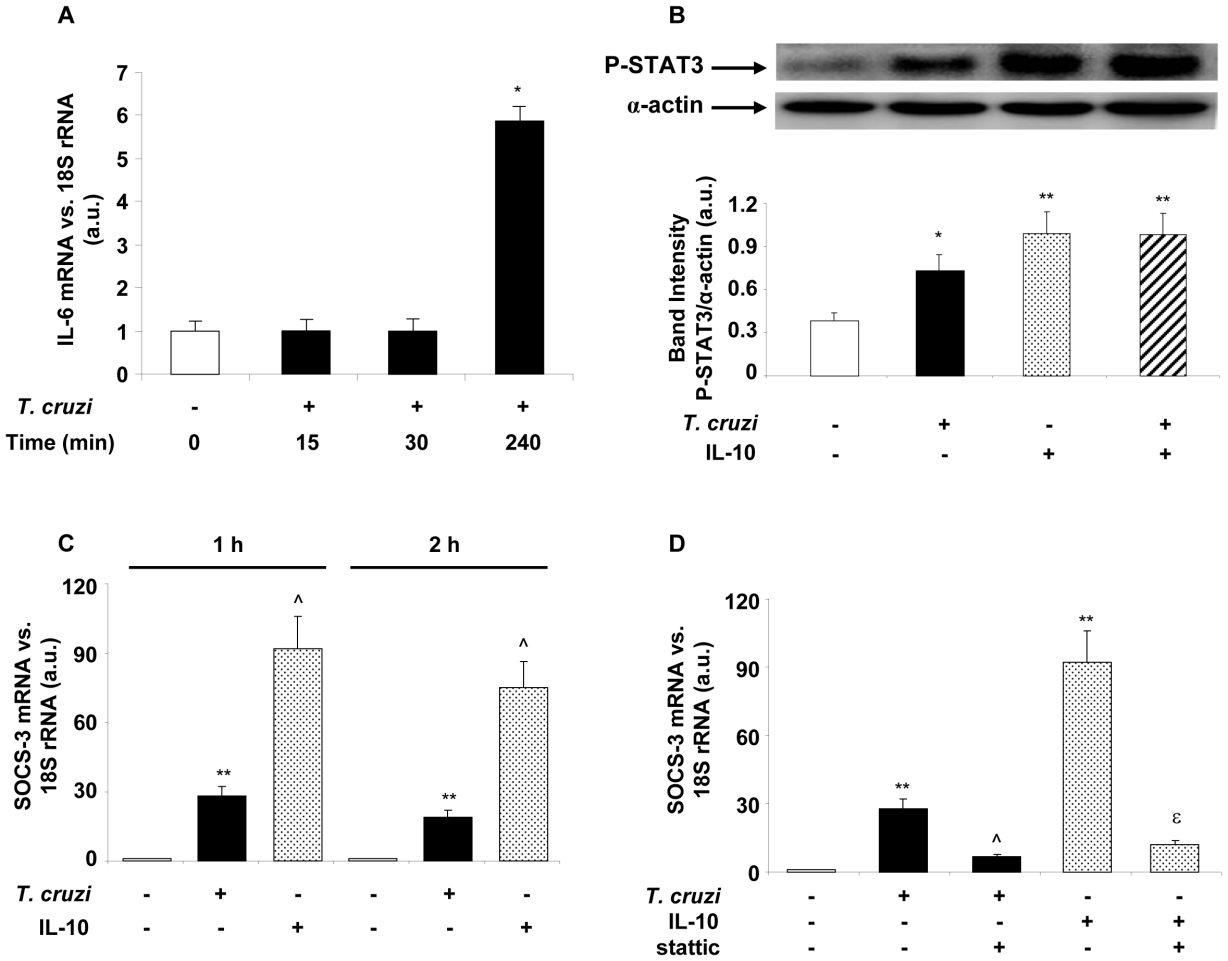
*T. cruzi* and IL-10 promote STAT3 activation and SOCS-3 expression. (A) Kinetics of IL-6 transcription after *T. cruzi* infection of cardiomyocytes. Cells were infected with *T. cruzi* trypomastigotes and at different time points mRNA for IL-6 was assessed using Q-RT-PCR. (B) Cells were infected or treated with IL-10 (20 ng/ml) or pre-treated with IL-10 and infected for 30 min and STAT3 activation was analysed by Wb. (C) Neonatal cardiomyocytes were infected or treated with IL-10 (20 ng/ml) for 1 or 2 h, and SOCS-3 mRNA levels were analysed by Q-RT-PCR. (D) Cells were pre-incubated with STAT3 inhibitor, stattic (10 µM), for 30 min before 1 h of *T. cruzi* infection or IL-10 treatment and SOCS-3 mRNA levels were analysed by Q-RT-PCR. For A, C and D, results were normalized against 18S and represent the mean±SD of three independent experiments. For B, results show the mean±SD (n = 3) and protein levels were normalized against α-actin. **p<0.01 vs. control cells; *p<0.05 vs. control cells; ˆp<0.01 vs. *T. cruzi* infected cells; ^ε^p<0.01 vs. IL-10 treated cells.

Taking into consideration that cytokines can activate STAT proteins, we sought to determine whether STAT3 transcription factor had a role in the signaling pathway that is initiated by *T. cruzi* infection and by a pleiotropic cytokine like IL-10. However, endogenous IL-10 was undetectable at the protein (ELISA) and mRNA (Q-RT-PCR) levels. Therefore, we tested the kinetics of IL-6 at the transcription level using Q-RT-PCR to test whether this cytokine was responsible for STAT3 activation. We detected IL-6 mRNA at 4 h after *T. cruzi* infection (Figure 2A). STAT3 activation was evaluated by the detection of the phosphorylated form of STAT3 by Western blot. These results indicate that both the infection with *T. cruzi* and IL-10 treatment are capable of activating STAT3 as early as 30 min after infection, since in both cases we detected the P-STAT3 form (Figure 2B). It has been suggested that, in response to cytokine signaling or to microorganisms, STAT3 activates SOCS-3 proteins involving the latter in the attenuation of pro-inflammatory signaling [17], [20]. Thereby, we speculated that SOCS-3 might have a role in the IL-10-mediated down-regulation of the pro-inflammatory response. Interestingly, an increase in SOCS-3 mRNA level was observed at 1 h or 2 h with either *T. cruzi* or IL-10, being the latter significantly greater (Figure 2C). We also determined a direct correlation between STAT3 activation and SOCS-3 expression, since in the presence of stattic, an inhibitor of STAT3, SOCS-3 expression was prevented both in infected cells and in those treated with IL-10 (Figure 2D).

### IL-10 Attenuates NF-κB and ERK Activity through SOCS-3

**Figure 3:**
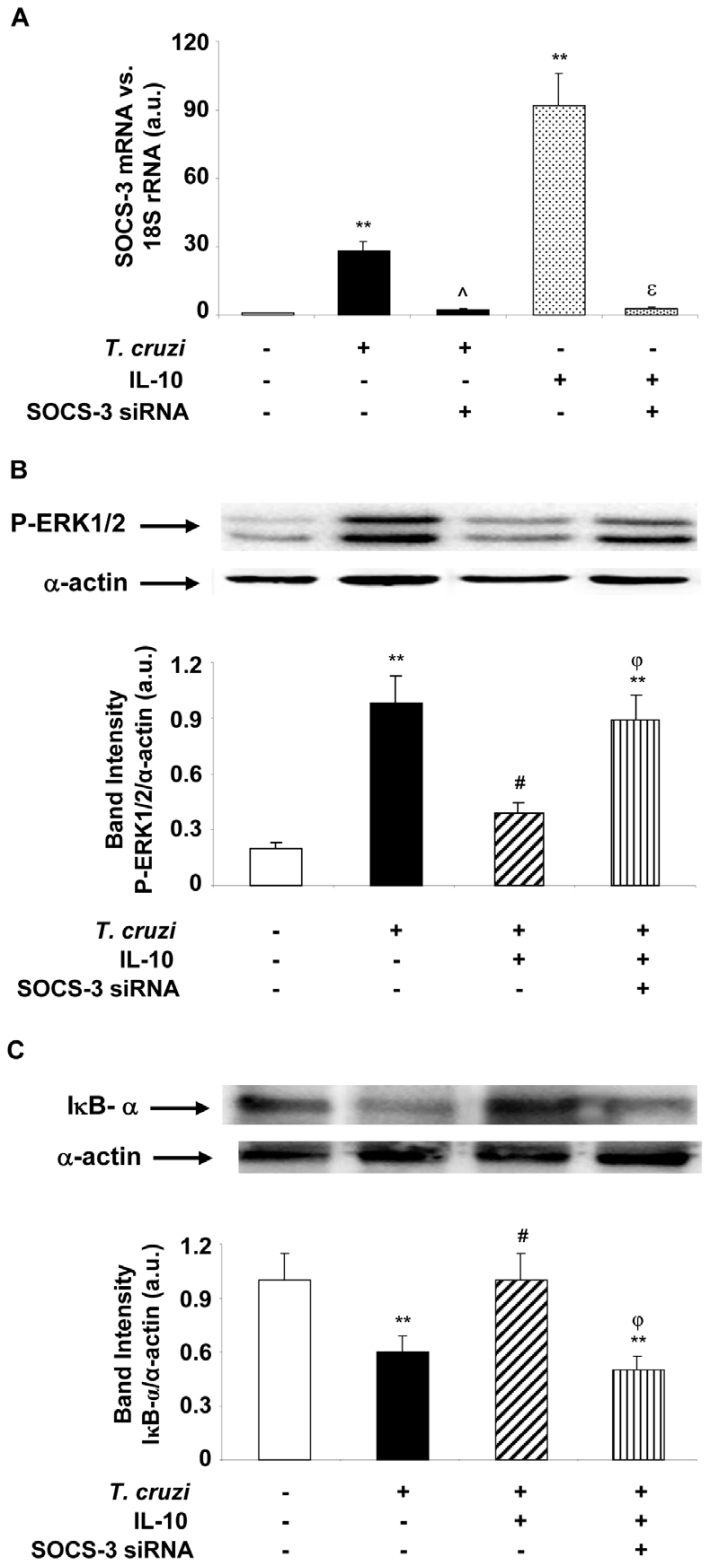
SOCS-3 is involved in the inhibition of ERK1/2-MAPK and NF-κB after IL-10 treatment of infected cardiomyocytes. (A) Cells were transfected with SOCS-3 siRNA for 72 h, infected with *T. cruzi* or treated with IL-10 for 1 h and SOCS-3 mRNA levels were analysed by Q-RT-PCR. (B) Cells were infected with *T. cruzi*, pre-treated with IL-10 or transfected with SOCS-3 siRNA for 72 h or pre-treated with IL-10 and infected for 20 min. P-ERK1/2-MAPK levels were determined by Wb with specific antibodies. (C) Cytosolic extracts from cardiomyocytes in the same conditions as in B but infected for 30 min were analysed by Wb to determine IκB-α expression. For A, results were normalized against 18S and represent the mean±SD of three independent experiments. For B and C, results show the mean±SD (n = 3) and protein levels were normalized against α-actin. **p<0.01 vs. control cells; ˆp<0.01 vs. *T. cruzi* infected cells; ^#^p<0.05 vs. *T. cruzi* infected cells;^ ε^p<0.01 vs. IL-10 treated cells; ^φ^p<0.05 vs. *T. cruzi* infected+IL-10 treated cells.

Given that IL-10 increases SOCS-3 levels and inhibits both *T. cruzi*-induced NF-κB and ERK activities, we postulated that SOCS-3 is the intermediary through which IL-10 acts in this experimental model. To corroborate this hypothesis, we silenced SOCS-3 expression by using SOCS-3-specific siRNA and evaluated the role of IL-10 on NF-κB and ERK activities in *T. cruzi*-infected cardiomyocytes. First, we performed Q-RT-PCR assays 72 h after transfection with SOCS-3-siRNA to evaluate the silencing efficiency. Figure 3A shows that the expression of SOCS-3 evidenced after *T. cruzi* infection and IL-10 treatment was silenced in transfected cells. In order to evaluate ERK1/2 and NF-κB activation pathways, cells were transfected with SOCS-3-siRNA, infected and pretreated with IL-10. Western blotting showed that SOCS-3 silencing prevented the inhibitory effect of IL-10 on the phosphorylation of ERK1/2 (Figure 3B). Cells transfected with SOCS-3-siRNA and treated with IL-10 showed IκB-α levels similar to those observed after infection, indicating that SOCS-3 is required for IL-10 inhibition of NF-κB (Figure 3C).

### IL-10 Inhibits Inflammatory Mediators in *T. cruzi*-infected Cardiomyocytes

**Figure 4:**
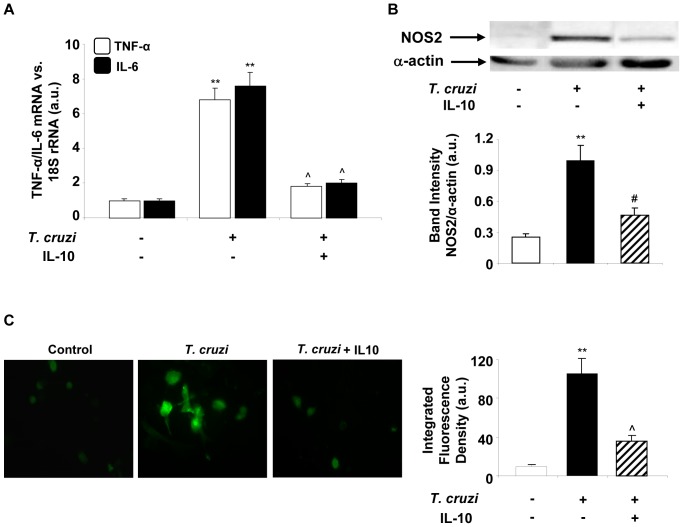
IL-10 treatment inhibits pro-inflammatory cytokines and NOS2 expression and activity in *T. cruzi* infected cardiomyocytes. (A) Cells were infected with *T. cruzi* or pre-treated with IL-10 (20 ng/ml) and TNF-α and IL-6 mRNA levels were analysed by Q-RT-PCR 4 h post infection. (B) NOS2 expression was analysed by Wb in total protein extracts obtained after 48 h of *T. cruzi* infection or after pre-treatment with IL-10. (C) Cells were grown on round glass cover slips and infected with *T. cruzi* or pre-treated with 20 ng/ml of IL-10 for 15 min. After 48 h of infection, NO synthesis was detected *in situ* by the NO indicator DAF-FM and visualized by fluorescence microscopy. For A, results were normalized against 18S and represent the mean±SD of three independent experiments. For B, results show the mean±SD (n = 4) and protein levels were normalized against α-actin. In C, microphotographs (400X) are representative of three independent experiments performed. The graph represents the mean±SD of the integrated fluorescence densities. **p<0.01 vs. control cells; ˆp<0.01 vs. *T. cruzi* infected cells; ^#^p<0.05 vs. *T. cruzi* infected cells.

Since IL-10 is involved in the control of the inflammatory response, we decided to study the effects of exogenous IL-10 addition on inflammatory mediators. Previous results have shown that cardiomyocytes are able to produce cytokine and NOS2 expression after *T. cruzi* infection [3]. When cardiomyocytes were pre-incubated with IL-10 (20 ng/ml), Q-RT-PCR results showed a significant decrease in IL-6 and TNF-α mRNA levels after 4 h of infection (Figure 4A). Western blotting assays also revealed that IL-10 is capable of inhibiting NOS2 protein expression in *T. cruzi*-infected cells (Figure 4B). In addition, we evaluated the *in situ* synthesis of NO by using 4-amino-5-methylamino-2′,7′-difluoro-fluorescein (DAF-FM), a highly sensitive indicator of the presence of NO. This reagent is virtually non-fluorescent until it reacts with NO, forming the fluorescent benzotriazole. Cardiomyocytes infected with *T. cruzi* for 48 h showed intense fluorescence, whereas those treated with IL-10 showed no fluorescent label (Figure 4C), indicating a clear inhibition of NO production.

**Figure 5:**
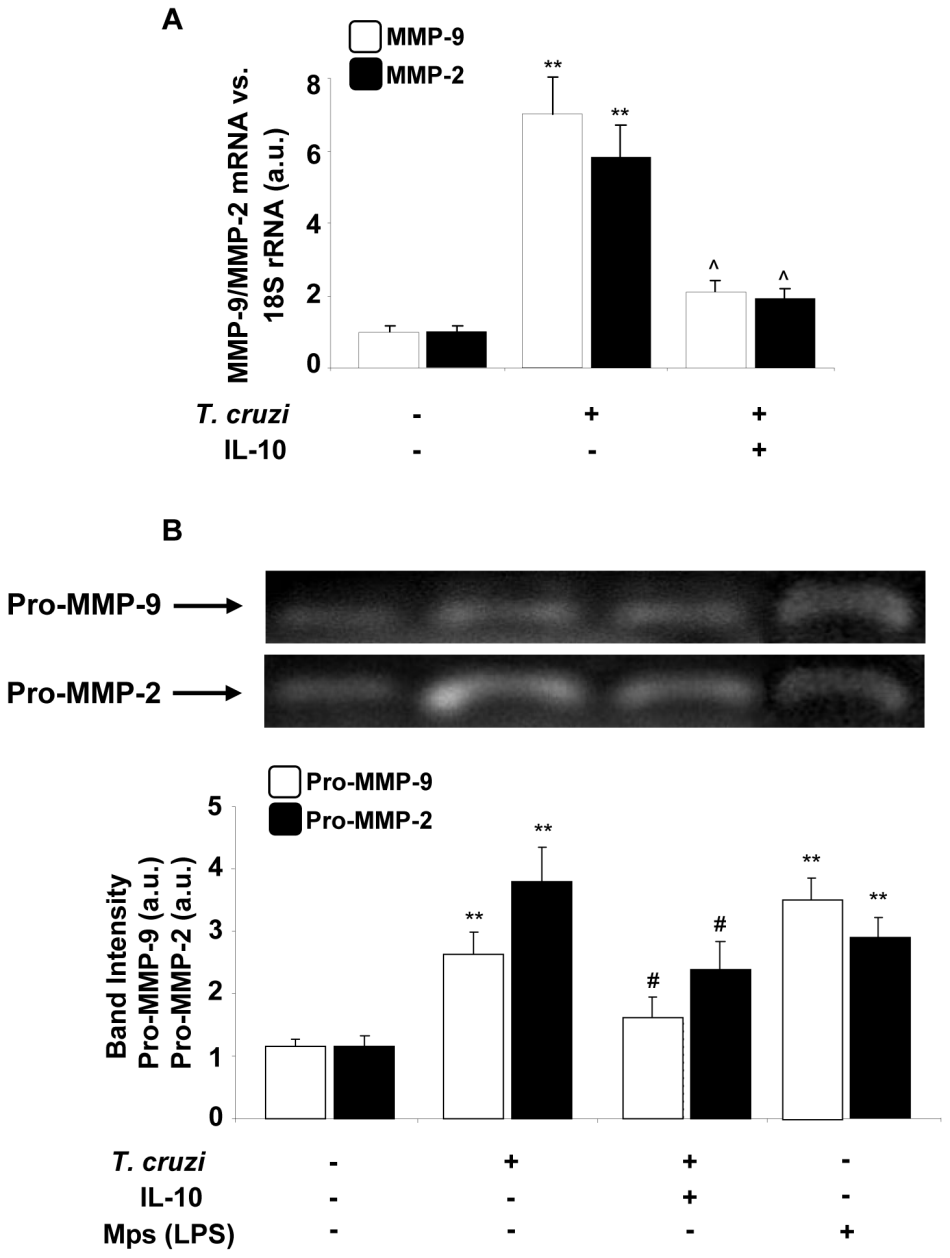
Effect of IL-10 treatment on cardiac matrix metalloproteinase (MMP)-9 and MMP-2 expression and activity after *T. cruzi* infection. (A) Neonatal cardiomyocytes were treated with IL-10 (20 ng/ml) for 15 min followed by 48 h of *T. cruzi* infection. MMP-9 and MMP-2 mRNA levels were analysed by Q-RT-PCR. (B) Under the same conditions as in A, zymograms of MMP-9 and MMP-2 activity were carried out in culture media of neonatal cardiomyocytes after 72 h of *T. cruzi* infection. White bars indicate the values of MMP-9 activity and black bars indicate the values of MMP-2 activity. Peritoneal macrophages (Mps) were stimulated with LPS for 24 hours and used as a positive control for MMP. In A, the results were normalized against 18S and represent the mean±SD of three independent experiments. For B, results represent the relative densitometric analysis of the activities of Pro-MMP-9 and Pro-MMP-2 of four experiments performed. **p<0.01 vs. Control cells; ˆp<0.01, ^#^p<0.05 vs. *T. cruzi* infected cells.

Based on these results, we decided to investigate whether IL-10 also has some effect on MMPs expression induced in cardiomyocytes by *T. cruzi* infection. For this purpose, we used Q-RT-PCR to evaluate MMP-9 and MMP-2 expression in cardiomyocytes infected and treated with IL-10. Figure 5A shows that IL-10 (20 ng/ml) inhibited the expression of both MMPs significantly compared with enzyme levels induced after 48 h of infection with *T. cruzi*. To evaluate whether the inhibition of mRNA expression exerted by IL-10 correlated with the activity of these enzymes, supernatants of cultured cardiomyocytes treated or not with IL-10 and infected for 72 h were tested by zymography. IL-10 significantly inhibited the gelatinolytic activity of proenzymes MMP-9 and MMP-2 induced by infection with *T. cruzi* in cardiomyocytes (Figure 5B).

### SOCS-3 is Involved in the Anti-inflammatory Effects Exerted by IL-10 in *T. cruzi*-infected Cardiomyocytes

**Figure 6:**
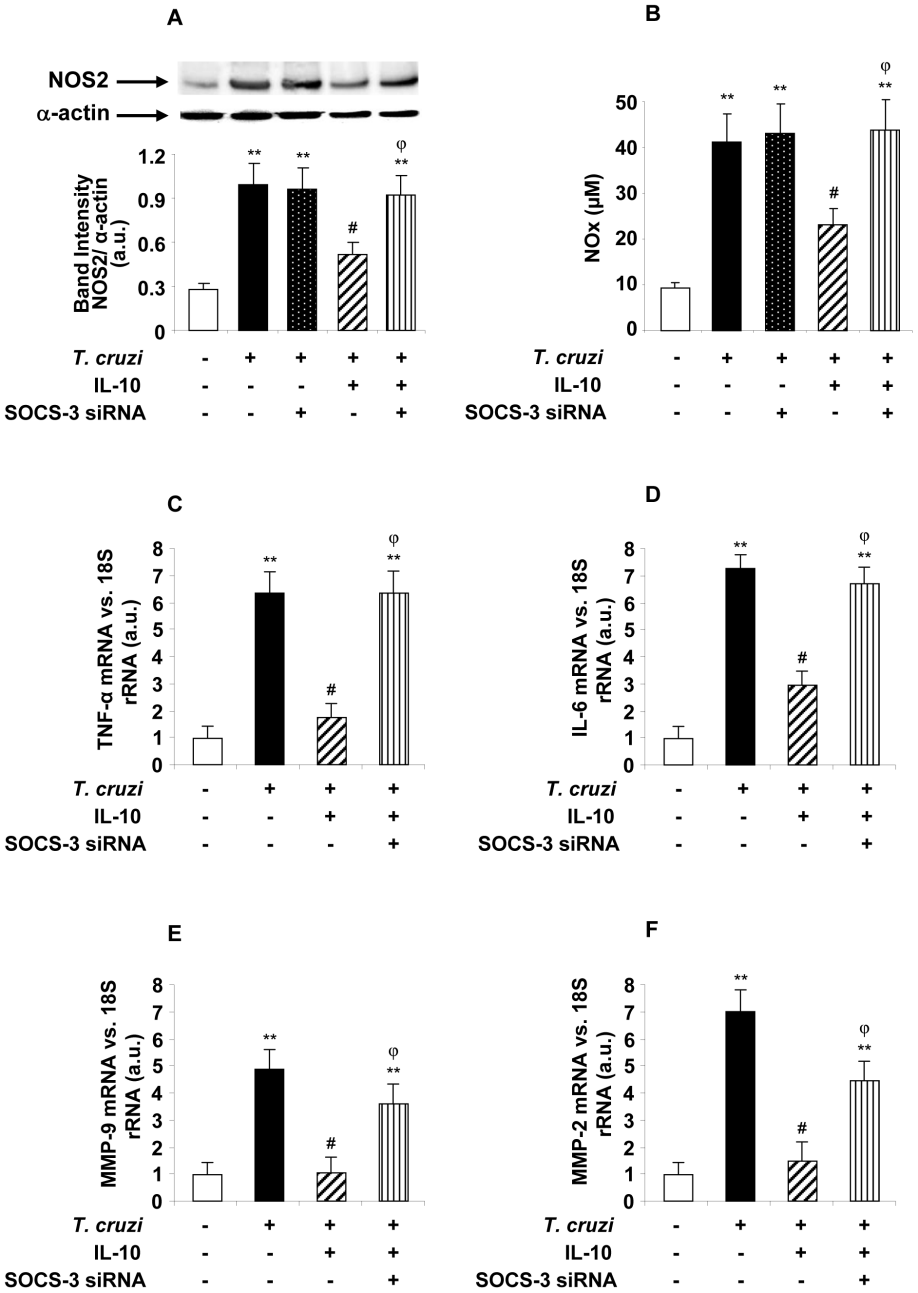
SOCS-3 is involved in the anti-inflammatory effects exerted by IL-10. Cardiomyocytes were pre-treated with 20 ng/ml of IL-10, infected with *T. cruzi* or transfected with SOCS-3 siRNA for 72 h, pre-treated with IL-10 and infected for 48 h. (A) NOS2 expression levels were determined by Wb. (B) In the same conditions NOS2 activity was evaluated by quantification of NO levels by Griess reaction in the culture media. (C) and (D) TNF-α and IL-6 mRNA levels were analysed by Q-RT-PCR in the same conditions but only for 4 h post infection. (E) and (F) In the same conditions as in (A), MMP-9 and MMP-2 mRNA levels were analysed by Q-RT-PCR. For A, results show the mean±SD (n = 3) and protein levels were normalized against α-actin. In B, results are expressed as mean±SD (n = 3). In C,D,E and F, the results were normalized against 18S and represent the mean±SD of three independent experiments **p<0.01 vs. control cells; ^#^p<0.05 vs. *T. cruzi* infected cells; ^φ^p<0.05 vs. *T. cruzi* infected+IL-10 treated cells.

Given that IL-10 enhances the expression of SOCS-3 and its silencing prevents ERK/MAPK and NF-κB inhibition of its activation, we sought to determine whether SOCS-3 also participates in the inhibition of inflammatory mediators in infected cells pre-treated with IL-10. Therefore, cells were treated with IL-10 and infected or transfected with SOCS-3-siRNA, and NO production as well as NOS2, cytokines, MMP9 and MMP2 expression were evaluated. Figure 6A and 6B show that when SOCS-3 was silenced in cardiomyocytes, IL-10 was unable to exert its modulatory effects on NOS2 expression or NO production. Similarly, in conditions of silencing of SOCS-3, IL10 was uncapable to inhibit the expression of TNF-α and IL-6 (Figure 6C and 6D). This was also found regarding the expression of MMP-9 and MMP-2, as shown in Figure 6E and 6F. No significant differences were found in the pro-inflammatory responses of infected cardiomyocytes, either silenced or not in the absence of IL-10 treatment (Data not shown). These results confirm that the STAT3/SOCS-3 pathway participates in the regulatory effects of IL-10 on *T. cruzi-*infected cardiomyocytes.

### SOCS-3 Silencing Reverts IL-10-induced Cardiomyocytes Parasitism

**Figure 7:**
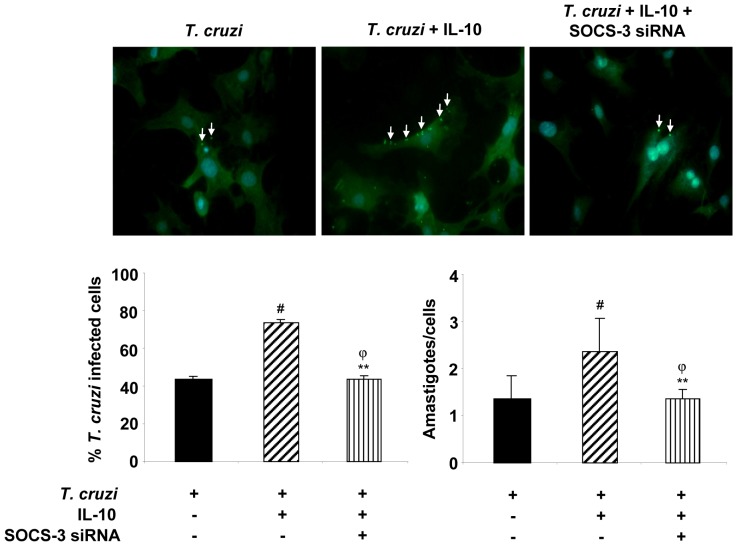
SOCS-3 silencing reverts IL-10-induced cardiomyocytes parasitism. Cells grown on glass coverslips were infected, infected and treated with 20/ml of IL-10 or silenced by siRNA for 72 h and then pre-treated with IL-10 and infected for 48 h. Infected cells were incubated with rabbit polyclonal serum against to *T. cruzi* followed by FITC-labeled goat anti-rabbit IgG. Afterwards, cells were counterstained with DAPI (300 nM). The percentage of infected cells (left bar graph) and the number of amastigotes/cell (right bar graph) are shown. Results represent the Mean ± SD of two experiments. Microphotographs are representative of 30 fields taken at 400X magnification. White arrows show FITC-anti-*T.cruzi-*labelled intracellular amastigotes.

In light of the effects of IL-10 on the pro-inflammatory response of cardiomyocytes during *T. cruzi* infection and the reversion of these effects by SOCS-3 silencing, we decided to determine the effects of this cytokine and SOCS-3 silencing on the percentage of infected cells and on the parasite load. Cells grown on glass coverslips were infected, infected and treated with IL-10 or silenced by siRNA and then infected and treated with IL-10. As expected, due to the inhibition of the anti-parasitic effector mechanisms exerted by IL-10, increased percentage of infected cells and number of amastigotes/cell were observed in IL-10-treated cardiomyocytes (Figure 7). Silencing of SOCS-3 with siRNA reverted this effect. Silencing of SOCS-3 did not modify *T. cruzi* parasitism in IL-10-untreated cells (data not shown).

## Discussion

In this study, we investigated the ability of IL-10 to inhibit NF-κB and ERK/MAPK pathways and consequently the production of pro-inflammatory mediators through the up-regulation of SOCS-3 expression in *T. cruzi-*infected cardiomyocytes.

During the acute phase of *T. cruzi* infection, cardiomyocytes, which produce cytokines such as IL-1β, IL-6 and TNF-α and mediators like NO, play an important role in the development of local inflammatory response [3]. In the early 1990s, it was described that the production of TGF-β and IL-10 are key to the inhibition of increased levels of IFN-γ and their damaging effects induced in response to *T. cruzi* infection both *in vitro* and *in vivo* experimental models [26], [27]. Later on, it was shown that IL-10 knockout mice are more susceptible to infection and have higher mortality rates than wild-type mice [28], [29]. Furthermore, it has been reported that a deficiency in the regulatory mechanisms of the inflammation in chagasic patients, either by lack of IL-10 production or excessive IFN-γ, could contribute to the pathogenesis of the disease [30]. In this regard, it has been reported that peripheral blood mononuclear cells isolated from patients in the indeterminate phase of Chagas’ disease produce higher levels of IL-10 than those isolated from patients with chronic cardiomyopathy [31], [32].

Based on these records, we investigated the role of IL-10 in our model of *T. cruzi-*infected cardiomyocytes with the lethal RA strain. We assessed the endogenous synthesis of IL-10 in primary cultures of infected cardiomyocytes, but could neither detect its expression by Q-RT-PCR assays nor in the supernatants using a commercial ELISA kit (data not shown). These results are consistent with those of Ponce NE. et al. [16], who described that isolated cardiomyocytes infected with *T. cruzi* do not produce detectable amounts of IL-10. However, in *in vivo* models of infection with *T. cruzi*, the cardiac expression of IL-10 has been associated with the extent of the inflammatory infiltrates present in the heart [33], [34]. In a previous work, we detected significant levels of IL-10 in the sera from mice infected with the RA and K98 strains of *T. cruzi* with different lethality [4]. These antecedents led us to evaluate the effects of the exogenous addition of IL-10 in cultures of infected cardiomyocytes. The present results show inhibition of NOS2 expression, by Western blotting and activity, using Griess assays, as well as using 4-amino-5-methylamino-2 ′,7′-difluorofluorescein (DAF-FM), an indicator of *in situ* NO production. We also observed that treatment with IL-10 significantly inhibited the expression of TNF-α and IL-6 induced by infection. Several factors contribute to chagasic cardiomyopathy and tissue damage, including parasite persistence in heart tissue [35]. The MMPs family may also contribute to the evolution of chagasic cardiomyopathy, facilitating parasitic infection and tissue remodeling. Furthermore, increased activity of cardiac MMP-2 and MMP-9 has been associated with mortality during the acute phase of *T. cruzi* infection, suggesting an important role in the induction of chagasic acute myocarditis [36]. In previous works, we have demonstrated that cardiomyocytes are able to express MMPs after LPS challenge or *T. cruzi* infection in different experimental models [3], [4], [23], [37]. In the present work, we found that pre-treatment with IL-10 inhibited MMP-2 and MMP-9 expression and activity after *T. cruzi* infection.

It has been described that the modulatory effect of IL-10 in the inflammatory response is associated with the inhibition of the NF-κB pathway [38]–[40]. Our results show that IL-10 treatment promoted less degradation of cytosolic IκB-α and reduced the translocation of p65 to the nucleus in infected cardiomyocytes compared with the untreated cells. Given that *T. cruzi* infection promotes ERK/MAPK activation, we also evaluated the role of IL-10 in infected cardiomyocytes and observed a decrease in phosphorylation levels of ERK1/2, indicating the inhibition of the activity of this pathway.

This study also provides evidence that phosphorylation of STAT3 contributes to the inflammatory response. In this regard, it has been shown that treatment with IL-10 in a murine model of myocardial infarction inhibits inflammatory parameters and participates in the cardiac remodeling process via activation of STAT3 [41]. Our results regarding the precedence of *T. cruzi-*dependent mechanisms over cytokine effects in the activation of STAT3 are in agreement with those of Stahl et al. [42] and Ponce et al [16] who show early activation of STAT3 upon infection of cardiomyocytes with *T. cruzi* trypomastigotes. While in the model of Ponce et al. [16] the anti-apoptotic effect of cruzipain is also responsible for IL-6 production, in our model the *T. cruzi* factor(s) responsible for STAT3 activation prior to IL-6 production remain to be elucidated.

It has been described that phosphorylated STAT3 is dimerized and induces nucleus transcription of the SOCS-3 gene [43]. There is some evidence for the participation of this protein in the mechanism by which IL-10 modulates the inflammatory response in several models. For example, SOCS-3 has been reported as a key mediator in the inhibitory effects of IL-10 in macrophages stimulated with LPS [44]–[46] and in an *in vivo* model of collagen-induced arthritis [47]. Recently, it has been reported that SOCS-3 acts as an endogenous inhibitor of pathological angiogenesis in mouse models of cancer and proliferative retinopathy [42]. Moreover, macrophages infected with *M. tuberculosis* show inhibition of NF-κB activation and induction of SOCS-3 expression as a mechanism of bacterial evasion of the inflammatory response [20]. Furthermore, the availability of knockout mice for SOCS-2 has revealed that this protein is an important regulator of the immune response generated in a acute model of *T. cruzi* infection [21]. However, the relevance of SOCS-3 expression in the anti-inflammatory effects exerted by IL-10 in *T. cruzi* infections remains to be investigated. In this work, we report that *T. cruzi* and IL-10 can up-regulate SOCS-3 expression through the activation of STAT3. However, the results demonstrate that SOCS-3 expression was higher in cardiomyocytes treated with IL-10. Indeed, STAT3 inhibition by stattic, a specific inhibitor of STAT3 activation, prevented SOCS-3 expression, confirming that SOCS-3 is induced by IL-10/STAT3 in our model. To further evaluate the mechanisms involved in IL-10 anti-inflammatory effects, we investigated the consequences of the specific knock-down of SOCS-3, demonstrating that SOCS-3 is essential for IL-10 signaling, since its silencing prevented IL-10 inhibitory effect on the expression and activity of NOS2.

Regarding the use of exogenous IL-10 as a means of controlling overt inflammatory responses, its advantages and drawbacks must be taken under consideration. In an experimental model of chronic enterocolitis in IL-10 KO mice, ip administration of IL-10 to weanlings significantly impeded the development of inflammation. While this treatment was not curative in adult mice, it ameliorated the course of the disease reducing the overt inflammatory Th1 response, characterized by exacerbated expression of IFN-γ, TNF-α, IL-1β and NO, and the incidence of colorectal denocarcinoma [48]. In a study on the beneficial effects of IL-10 treatment in an experimental model of rheumatoid arthritis, Persson *et al* [49] showed similar effects for IL-10: it limited the development or reduced the ongoing effects of type II collagen-induced arthritis. Regarding studies in humans, results are more contradictory. In a study by van Roon et al. [50] treatment of 6 patients with IL-10 didn’t improve their clinical score. Also, Heo et al. [51] observed that, in patients with rheumatoid arthritis, both peripheral blood and synovial fluid CD4^+^ T cells produced more IL-17 and less IL-10 than those of healthy donors.

While treatment with IL-10 may be of significant aid in sterile (pathogen-free) inflammatory disorders, its use in inflammatory processes that arise from infections may represent a double edged sword. For this reason, careful evaluation of the administration route, dose and timing should be taken into consideration. Several studies regarding the role of IL-10 in the progression of experimental and human Chagas’ disease have been conducted. Some of them have shown that IL-10 mediates susceptibility to infection by *T. cruzi*. Reed et al. [52] showed that highly susceptible mice transcribed IL-10 mRNA early upon infection in contrast with those with less susceptibility. Moreover, anti-IL-10 treatment increased their survival.

Susceptibility to infection seems to be associated by the ability of *T. cruzi* to drive dendritic cells (DC) towards a regulatory IL-10-producing phenotype. Alba Soto et al. [53] showed that in IL-10 KO mice, DC are able to induce more potent Th1-type response both in mixed-leukocyte reactions as well as upon *T. cruzi* lysate stimulation of T cells *in vitro*, in comparison with wildtype mice. Again, as shown by Reed et al. [52] their findings suggest that early IL-10 production by infected mice contributes to *T. cruzi* susceptibility. Altogether, these results suggest that treatment with IL-10 during the acute stage of the disease might be disadvantageous and even deleterious. In spite of this, IL-10 may exert benefitial effects even during the acute stage of the disease. Guedes et al. [54] have shown in a dog model of Chagas’ disease evolution, that increased IL-10 expression during the acute infection with three different parasite strains (Y, ABC and Berenice-78) correlates positively with a better outcome during the chronic stage in terms of development of chronic cardiomyopathy. In the same line of evidence, Roffê et al. [55] using a mouse model of *T. cruzi* infection with the Colombiana strain have shown that IL-10 is required to avoid the inflammatory reaction that leads to fatal myocarditis. In their model C57BL/6J (H-2^b^) mice are resistant to fatal myocarditis in comparison with C3H/HeSnJ (H-2^k^) mice. Deletion of IL-10 gene in mice with the resistant background (H-2^b^) renders them susceptible to early death, similarly to the genetically susceptible mice (H-2^k^).

The evidence regarding the role of IL-10 in the evolution of human Chagas’ disease is less clear. This is mainly due to the fact that detected acute cases are immediately treated using the presently available trypanocidal drugs. Therefore, the data so far available come from studies in chronically infected individuals at the indeterminate or chronic stages of the disease. It has been shown that the frequency of Treg cells is augmented in patients with the indeterminate vs patients with the cardiac form of the disease. In this regard, de Araújo et al. [56] showed that the frequency of Treg (CD4^+^ CD25^high^ Foxp3^+^) T cells that produce IL-10 was higher in patients in the indeterminate phase than in patients with the cardiac form of the disease. Interestingly, patients with the higher frequency of IL-10-producing CD4^+^ CD25^high^ Foxp3^+^ also had the higher left ventricular ejection fraction, which is a good prognosis marker. The same group [57], also recently showed that patients with the indeterminate form of the disease had higher frequencies of IL-10- and IL-17-expressing Treg than patients with the cardiac form. Moreover, the latter had higher frequencies of IL-6+, IFN-γ+, TNF-α+ and CTLA-4+ Treg cells. On the other hand, in a recent study by de Melo *et al.* [58] the authors didn’t find any correlation between clinical stage and gene expression of either IL-10 or IFN-γ as measured by Q-RT-PCR, upon stimulation of peripheral blood mononuclear cells with *T. cruzi* recombinant antigens.

Altogether, the results coming from experimental models and from research in patients, suggest that a delicate balance between the pro- and anti-inflammatory responses during the acute infection, is required to preclude either uncontrolled parasite proliferation or the development of an overt inflammatory response leading to tissue damage that, initiated during the acute infection may result in chronic chagasic cardiomyopathy.

In summary, we show that IL-10 acts as a negative regulator of pro-inflammatory signaling by up-regulation of SOCS-3 in cardiomyocytes infected with *T. cruzi*. Our results provide new insights into the probable mechanism by which IL-10/STAT3/SOCS-3 negatively regulate NF-κB and ERK/MAPK–dependent gene expression of pro-inflammatory mediators. Since cardiomyocytes are a major target of *T. cruzi* infection, producing significant levels of NO and cytokines, the treatment with IL-10 using the proper timing, dose and route might exert beneficial effects on the heart, related to its anti-inflammatory effects in the acute phase of Chagas’ disease that, as discussed earlier, may determine the fate of an ongoing inflammatory response with, so far, unpredictable consequences.
